# Identification of traumatic brain injury induced molecular signatures using spatial transcriptomics

**DOI:** 10.1002/alz70861_108495

**Published:** 2025-12-23

**Authors:** Mehwish Anwer, Aditya Swaro, Brianna Bristow, Jianjia Fan, Wai Hang Cheng, Mark Cembrowski, Cheryl L Wellington

**Affiliations:** ^1^ University of British Columbia, Vancouver, BC Canada

## Abstract

**Background:**

Traumatic brain injury (TBI) results in multifaceted neuropathology including neuroinflammation, neuronal loss, axonal damage and blood brain barrier damage, which can lead to cognitive impairment and dementia including Alzheimer’s disease (AD) and related dementias (ADRDs). Detailed analysis of the molecular and cellular changes that occur in injured brain is crucial to understand TBI’s contributions to dementia and AD. The Closed Head Impact Model of Engineered Rotational Acceleration (CHIMERA) is a non‐surgical model of impact‐acceleration injury that mimics the biomechanics and pathophysiology of human TBI.

**Methods:**

Adult male mice received a mild CHIMERA injury (2.1J) and acute neurological deficits were assessed as compared to sham group. At 7 days post‐TBI, cardiac blood was collected for biomarker analysis and brain was harvested for 10x genomics Visium spatial transcriptomic and multiplexed fluorescence in situ hybridization (mFISH) analysis.

**Results:**

Injured mice showed delayed righting reflex recovery and poor performance on neurological tests. Increased plasma glial fibrillary acidic protein (GFAP) and neurofilament light levels (NfL) were found in TBI compared to sham mice. We identified widespread (6614 DEGs: 82% downregulated, 18% upregulated) as well as regional cell type specific gene dysregulation in several clusters. We cross‐referenced our dataset with genes identified in human genome wide association studies in AD and TBI and found conserved genes in all three datasets (*Ace, AdamTS4, AdamTS1, Trem2, ApoE, Cacna1a, RbFox1, Clu, Apoc1, Mapt*). In the optic tract cluster, we identified that the astrocytic marker *Gfap*, microglial marker *Aif1* and several disease‐associated microglia (DAMs) genes including *Apoe, Ctsd, Trem2* were upregulated. GFAP immunolabelling confirmed astrogliosis in optic tract and hippocampus. In the neocortical cluster, we found downregulation of *Itm2c*, a negative regulator of amyloid‐beta peptide production, and *Scg5*, a chaperone protein that prevent aggregation of secreted proteins associated with neurodegeneration, which may contribute to TBI‐associated susceptibility to AD. mFISH labelling validated downregulation of these genes in cortex.

**Conclusions:**

Our data‐rich spatial transcriptomics approach identified molecular and cellular substrates crucial to TBI pathology and its comorbidities including AD and ADRD. Together, this work will provide spatial molecular maps of diffuse brain injury and novel insights into TBI’s contributions to dementia.

